# Year-Round Thermal Error Modeling and Compensation for the Spindle of Machine Tools Based on Ambient Temperature Intervals

**DOI:** 10.3390/s22145085

**Published:** 2022-07-06

**Authors:** Xinyuan Wei, Honghan Ye, Xugang Feng

**Affiliations:** 1 School of Electrical and Information Engineering, Anhui University of Technology, Ma’anshan 243032, China; weixy@ahut.edu.cn; 2 Department of Industrial and Systems Engineering, University of Wisconsin—Madison, Madison, WI 53705, USA; hye42@wisc.edu

**Keywords:** CNC machine tool, thermal error, ambient temperature interval, model robustness

## Abstract

The modeling and compensation method is a common method for reducing the influence of thermal error on the accuracy of machine tools. The prediction accuracy and robustness of the thermal error model are two key performance measures for evaluating the compensation effect. However, it is difficult to maintain the prediction accuracy and robustness at the desired level when the ambient temperature exhibits strong seasonal variations. Therefore, a year-round thermal error modeling and compensation method for the spindle of machine tools based on ambient temperature intervals (ATIs) is proposed in this paper. First, the ATIs applicable to the thermal error prediction models (TEPMs) under different ambient temperatures are investigated, where the C-Means clustering algorithm is utilized to determine ATIs. Furthermore, the prediction effect of different numbers of ATIs is analyzed to obtain the optimal number of ATIs. Then, the TEPMs corresponding to different ATIs in the annual ambient temperature range are established. Finally, the established TEPMs of ATIs are used to predict the experimental data of the entire year, and the prediction accuracy and robustness of the proposed ATI model are analyzed and compared with those of the low and high ambient temperature models. The prediction accuracies of the ATI model are 20.6% and 41.7% higher than those of the low and high ambient temperature models, respectively, and the robustness is improved by 48.8% and 62.0%, respectively. This indicates that the proposed ATI method can achieve high prediction accuracy and robustness regardless of the seasonal temperature variations throughout the year.

## 1. Introduction

In the machining process, changes in the internal and external heat sources such as motor operation, friction, cutting heating, and environmental temperature result in thermal errors, which represent 40–70% of the total errors of machine tools [1,2]. With the development of high-precision CNC machine tools, the influence of thermal errors on the tool performance is gradually becoming dominant [3]. To reduce such influence on machine tools, there are two main approaches. The first approach is to establish the analytical model and then to simulate and analyze thermal error laws. Although numerical analysis is promising to compensate for thermal errors, it is extremely difficult for the numerical method to build an exact structural model in practice and simulate the thermal deformation of machine tools because of complicated deformation processes. Alternatively, software compensation methods are commonly used to reduce the influence of thermal errors on the accuracy of machine tools [4]. In this method, temperature sensors are first installed on various locations of a machine, and a thermal error prediction model is established based on the temperature information collected from those sensors. Then the established model is embedded into the CNC system to realize thermal error compensation in real-time. Such a method, which is generally established by selecting relevant temperature-related variables and designing an appropriate modeling algorithm, offers high prediction accuracy and robustness [5].

First, the selected temperature variables are commonly called temperature-sensitive points (TSPs) in the literature. Currently, the most common TSP-selection algorithm is fuzzy clustering combined with the grey correlation degree algorithm [6,7,8,9]. The algorithm mainly involves classifying temperature variables and selecting one variable from each class as a TSP that has the greatest correlation with the thermal error. Based on the concept of this algorithm, researchers have proposed the rough set theory combined with the grey correlation degree algorithm [10] and fuzzy clustering combined with the correlation coefficient algorithm [11] to select the TSPs. Second, the algorithms commonly used to establish the thermal error prediction model (TEPM) include the multiple linear regression algorithm [6,12,13], time series algorithm [14,15], neural network algorithm [7,8,10,13], and support vector machine algorithm [16,17].

Recently, researchers have further studied the thermal error modeling algorithm of CNC machine tools [18,19,20,21,22] to improve the accuracy and robustness of thermal error prediction. However, the thermal error data in these studies had very small variations of ambient temperature, and the influence of ambient temperature was rarely considered. Unfortunately, their models perform poorly when the ambient temperature changes significantly, where the ambient temperature has a non-negligible influence on the thermal error modeling and compensation [1,12]. In fact, a significant change in the environmental temperature would lead to changes in the law of thermal deformation of the key parts of a machine tool. To verify this observation, we conducted the analysis of variance (ANOVA) of different sets of spindle thermal error data throughout a year. The results indicated a significant difference between the spindle thermal error laws under different ambient temperatures, i.e., the spindle thermal error law of a machine tool changes significantly with the ambient temperature. Thus, the constructed TEPMs from previous studies are difficult to use in accurately predicting spindle thermal errors over a wide range of ambient temperatures. In this regard, Zhang et al. [23] and Li et al. [24] used the finite element method to study thermal deformation laws of machine tools under the condition of time-varying environmental temperature. This method can be used to obtain the accurate thermal deformation of a machine tool under different ambient temperatures. However, it is a complex method, and the established model is not universal. More recently, Liu et al. [25] constructed thermal error models on both sides of a segment point of the ambient temperature. However, they only considered one segment point in their model and did not investigate the optimal number of segment points. Therefore, there exists a research gap, and the existing literature lacks a methodology that systematically analyzes the influence of ambient temperatures on the predictions and provides high accuracy and robustness performances.

Different from the existing methods [9,18,19] that have not considered the ambient temperatures in their models, the main aim of the research in this paper is to systematically investigate the influences of different ambient temperatures on thermal error prediction. The objective of this paper is to provide a thermal error modeling method, which takes ambient temperatures into consideration, so that the prediction accuracy and robustness can be further improved. To fill the aforementioned research gap, in this paper, we propose a year-round thermal error modeling and compensation method for the spindle of machine tools based on ambient temperature intervals (ATIs). Specifically, first, the ATIs applicable to the TEPMs are investigated based on experimental data collected over an entire year. Second, the C-means clustering algorithm is utilized to determine the ATIs. The influence of the number of ATIs on the prediction effects is further analyzed to determine the optimal number of ATIs. Then, the TEPMs are established with the optimal number of ATIs in the annual ambient temperature range. Finally, the prediction effects of the established TEPMs based on ATIs are analyzed and compared with those of the low and high ambient temperature models using the annual experimental data. As a result, the proposed method has two main contributions: (1) Different from the simple segmentation in [25], the C-means clustering algorithm is embedded and generic to find the number of ATIs; (2) the proposed method based on the selected ATIs performs better than the models that do not consider ambient temperatures in terms of both prediction accuracy (over 20% improvements) and robustness (over 48% improvements). The proposed method provides an important reference for thermal error modeling and compensation for CNC machine tools in a large ambient temperature range, and it is of great significance for promoting the development and precision of intelligent manufacturing.

The remainder of this paper is organized as follows. Section 2 introduces the modeling algorithms used in this study. In Section 3, an analysis of the experimental data collected over one year is presented, including the significance test of thermal error difference and the mutual prediction effect analysis of the annual experimental data. Section 4 first introduces the overview of the proposed modeling and compensation method based on the ATIs, and then it provides the details of determining ATIs based on the C-Means clustering method and selecting the optimal number of ATIs; it finally analyzes the prediction effects of the proposed ATI method. Section 5 draws the conclusions along with their applications and discusses the future scope of the study.

## 2. Thermal Error Modeling Algorithms

The thermal error modeling theory generally includes TEPM establishment and TSP selection algorithms. Principal component regression (PCR) [26], ridge regression [20], and partial least squares [27,28] are the common partial regression algorithms that can effectively suppress the influence of collinearity between input variables. Based on our preliminary studies, we used the PCR algorithm to construct the TEPM and the correlation coefficient algorithm to select the TSPs, which are introduced in Section 2.1 and Section 2.2, respectively.

### 2.1. Thermal Error Prediction Model (TEPM) Establishment Algorithm

Principal component analysis transforms a group of correlated variables into a group of linearly uncorrelated variables using orthogonal transformation, where the transformed variables are called principal components. Then, the principal components are further used as independent variables to establish the regression model.

In this study, temperature data were assumed as $X=\left(x_{1},x_{2}\ldots x_{p}\right)$, where p represents the number of temperature variables. The specific steps for establishing the thermal error PCR model are as follows.

**Figure 1:**
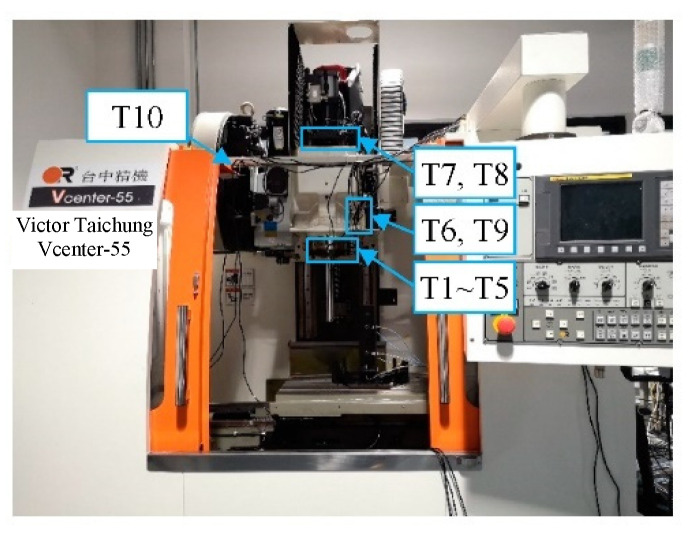
Experimental object.

1. Normalize the original data $X=\left(x_{1},x_{2}\ldots x_{p}\right)$ to obtain $X^{*}=\left(x_{1}^{*},x_{2}^{*}\ldots x_{p}^{*}\right)$. The standardized formula for this is as follows:

(1) xi*=xi−ExiVarxii=1,2,…p,

where original data $x_{i}$ is the temperature information on variable i collected from sensor i, and those sensors are installed in various locations of the machine tool, which will be shown later in Figure 1 and Table 1. In addition, $E\left(x_{i}\right)$ and $Var\left(x_{i}\right)$ are the expected value (average) and the variance of the $x_{i}$, respectively. Principal components $Z_{i}$ can be obtained from the eigenvalues and corresponding eigenvectors of a correlation coefficient matrix P of the original data $X=\left(x_{1},x_{2}\ldots x_{p}\right)$:

(2) $$\begin{array}{c} \left\{\begin{array}{c} Z_{1}=e_{11}*x_{1}^{*}+e_{21}*x_{2}^{*}+\ldots +e_{p1}*x_{p}^{*}\\ Z_{2}=e_{12}*x_{1}^{*}+e_{22}*x_{2}^{*}+\ldots +e_{p2}*x_{p}^{*}\\ \ldots \\ Z_{p}=e_{1p}*x_{1}^{*}+e_{2p}*x_{2}^{*}+\ldots +e_{pp}*x_{p}^{*} \end{array}\right., \end{array}$$

where $e_{i}={\left(e_{i1},e_{i2},\ldots ,e_{ip}\right)}^{T}$ is the eigenvector of the correlation coefficient matrix P, and each entry ($P_{i,j}$) of the matrix P can be calculated as follows:

$$P_{i,j}=\frac{Cov\left(x_{i},x_{j}\right)}{\sqrt{Var\left(x_{i}\right)Var\left(x_{j}\right)}},$$

where i=1, 2,…p, j=1, 2,…p, and $Cov\left(x_{i},x_{j}\right)$ is the covariance between $x_{i}$ and $x_{j}$. Note that all elements of the main diagonal of P (namely when i=j) have the value of one since the covariance of $x_{i}$ with itself is $Var\left(x_{i}\right).$

2. Calculate the cumulative contribution rate $V_{g}$ of the ith principal component; the formula is as follows:

(3) Vg=∑i=1gλi∑i=1pλig=1, 2, … p,

where $\lambda_{i}$ is the eigenvalue of matrix P corresponding to eigenvector $e_{i}$. In this study, the principal components with a $V_{g}$ of more than 95% were selected as TSPs to avoid a loss of information.

3. Normalize the original data of thermal error y using Equation (1) to obtain $y^{*}$ and then establish the regression model between $y^{*}$ and the selected principal components:

(4) $$\begin{array}{c} {\hat{y}}^{*}={\hat{a}}_{1}Z_{1}+{\hat{a}}_{2}Z_{2}+\ldots +{\hat{a}}_{g}Z_{g}, \end{array}$$

where ${\hat{a}}_{1},\ldots ,{\hat{a}}_{g}$ are the coefficients of the regression model. Specifically, vector $\hat{a}={\left({\hat{a}}_{1},\ldots ,{\hat{a}}_{g}\right)}^{T}$ can be obtained by calculating $Z^{T}Z^{-1}Z^{T}y^{*}$, where $Z=\left(Z_{1},\ldots ,Z_{g}\right)$, since both Z and $y^{*}$ are known. Please note that the intercept is not included here since all data are centered with mean 0 by normalization.

4. Substitute the formula of each principal component in Equation (2) into regression Equation (4) to obtain the regression equation between standardized variables $x_{1}^{*},x_{2}^{*}\ldots x_{p}^{*}$ and dependent variable $y^{*}$:

(5) $$\begin{array}{c} {\hat{y}}^{*}={\hat{\beta }}_{1}x_{1}^{*}+{\hat{\beta }}_{2}x_{2}^{*}+\ldots +{\hat{\beta }}_{p}x_{p}^{*}, \end{array}$$

where ${\hat{\beta }}_{i}=\left({\hat{a}}_{1},\ldots ,{\hat{a}}_{g}\right){\left(e_{i1},\ldots ,e_{ig}\right)}^{T}$ for i=1, 2,…p. The regression model between the original variables can be obtained from the relationship between estimated parameter ${\hat{\beta }}_{i}$ in Equation (5) and the original data regression model estimated parameter ${\hat{b}}_{i}$:

(6) $$\begin{array}{c} \hat{y}={\hat{b}}_{0}+{\hat{b}}_{1}x_{1}+{\hat{b}}_{2}x_{2}+\ldots +{\hat{b}}_{p}x_{p}, \end{array}$$

where the relationship between ${\hat{\beta }}_{i}$ and ${\hat{b}}_{i}\;$ is as follows.

(7) $$\begin{array}{c} \left\{\begin{array}{c} {\hat{b}}_{i}={\hat{\beta }}_{i}*\frac{\sqrt{Var\left(y\right)}}{\sqrt{Var\left(x_{i}\right)}}\\ {\hat{b}}_{0}=E\left(y\right)-\overset{p}{\underset{i=1}{\sum }}{\hat{b}}_{i}*E\left(x_{i}\right) \end{array}\right. \end{array}$$

### 2.2. TSP Selection Algorithm

As the PCR algorithm can suppress the influence of collinearity between input variables, the temperature variables did not have to be classified to reduce the correlation among the TSPs. We used Pearson’s correlation coefficient to determine the relationship between the temperature variable and thermal error. The temperature variables with high correlation coefficients were selected as the TSPs. The equation of the Pearson correlation coefficient is as follows:

(8) $$\begin{array}{c} \rho_{x_{i}y}=\frac{Cov\left(x_{i},y\right)}{\sqrt{Var\left(x_{i}\right)Var\left(y\right)}}, \end{array}$$

where $\rho_{x_{i}y}$ is the correlation coefficient between the ith temperature variable $x_{i}$ and the thermal error y. $Cov\left(x_{i},y\right)$ is the covariance between $x_{i}$ and y. $Var\left(x_{i}\right)$ and $Var\left(y\right)$ are the variances of $x_{i}$ and y, respectively. The equations are as follows:

(9) $$\begin{array}{c} Cov\left(x_{i},y\right)=\frac{\sum_{k=1}^{n}\left(x_{i,k}-E\left(x_{i}\right)\right)\left(y_{i}-E\left(y\right)\right)}{n-1}, \end{array}$$

(10) $$\begin{array}{c} Var\left(x_{i}\right)=\frac{\sum_{k=1}^{n}{\left(x_{i,k}-E\left(x_{i}\right)\right)}^{2}}{n-1}, \end{array}$$

where n is the number of observations/samples in the data.

## 3. Experimental Setup and Data Analysis

Thermal error measurement experiments were conducted on a three-axis vertical machining center for one year. In this section, an analysis of variance was performed on each batch of the experimental data. Then, the correlation coefficient and PCR algorithm were used to establish the TEPM. The prediction effects of the established models were further analyzed to prove that it was difficult to maintain their robustness over a large range of ambient temperatures.

### 3.1. Thermal Error Measurement Experiments

**Figure 2:**
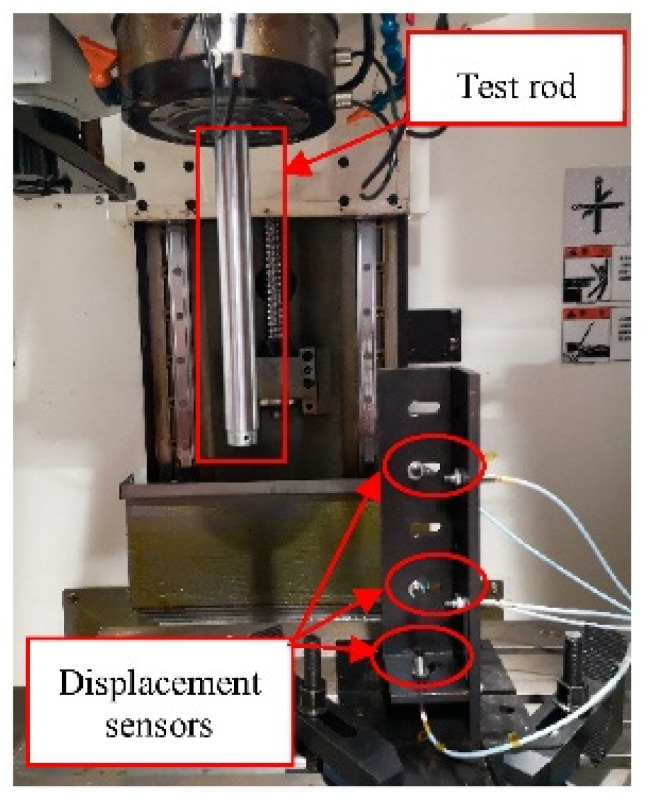
Distribution of displacement sensors.

The annual thermal error experimental object was a Vcenter-55 three-axis vertical machining center (Figure 1). The five-points measurement method was used for the spindle thermal error measurement according to the International Standard “Test code for machine tools—Part 3: Determination of thermal effects” (ISO 230-3:2020 IDT) [29]. Two displacement sensors each were used for the X and Y directions, and another one in the Z direction (Figure 2).

High-precision capacitance sensors were used to measure the spindle thermal error and their measurement accuracy was 1 μm. Ten Pt resistance temperature sensors were used to measure the temperature change at different positions of the machine tool. These temperature sensors are used to measure the main heat sources of the machine tool, such as spindle motor, spindle bearing, and so on. Ambient temperature is also measured by the sensor located at the machine tool frame. The distribution of the 10 temperature sensors is shown in Figure 1. The measurement accuracy of the temperature sensors was 0.1 °C, and their installation sites and measurement functions are listed in Table 1.

According to the International Standard [29], the spindle of the machine tool should idle at constant speed, and the worktable should reciprocate at a constant feed speed along the X- and Y-axes. Temperature and thermal error data were collected every 5 min for more than 4 h. In the annual experiment, the spindle speeds were set to 2000, 4000, and 6000 rpm, and the feed speed to 1500 mm/min. Each batch of experiments was performed after cooling the machine tool for 2 days.

### 3.2. Experimental Data Analysis

**Figure 3:**
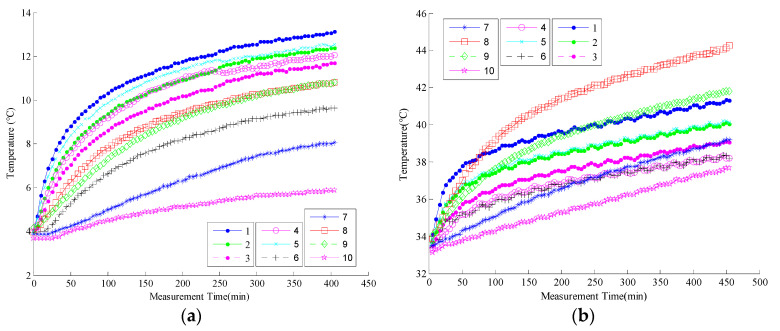
(**a**) Temperature change curves of experimental batches K1; (**b**) temperature change curves of experimental batches K46.

A total of 46 batches of effective experimental data were collected over an entire year and were sorted according to the initial ambient temperature recorded as K1–K46. Figure 3 graphically plots the temperature measurement results of the K1 experiment, which was conducted in winter, with the lowest initial ambient temperature and K46, which was conducted in summer, with the highest initial ambient temperature. As observed, the temperature change curves of K1 and K46 have significant differences.

**Figure 4:**
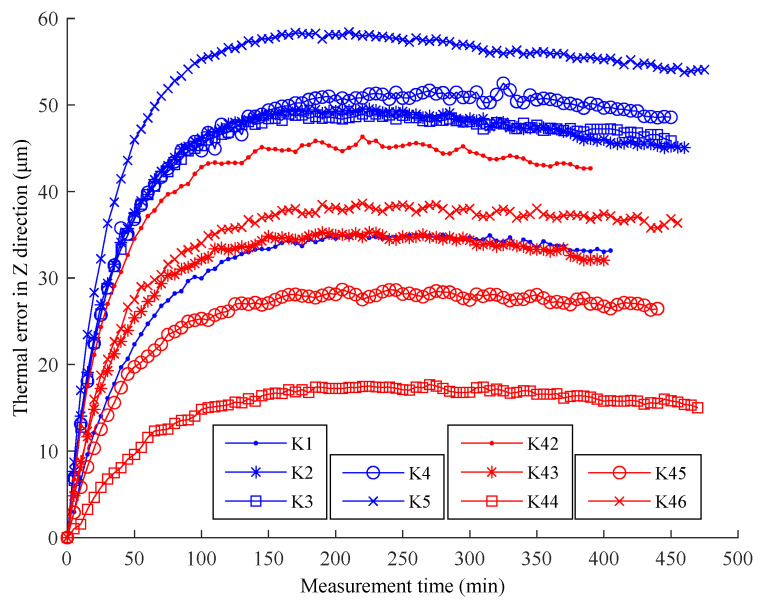
Thermal error curves of ten batches of experiments.

The thermal error curves of the 46 batches can be drawn similarly. Only the thermal error curves of batches K1–K5 and K42–K46 could be drawn, due to the large volume of data (Figure 4). Figure 4 reveals significant differences in the thermal error law between the low and high ambient temperature experiments. The thermal error in the low temperature environment was larger because its gradient began to increase by a greater margin after the machine tool had run for a certain time.

**Figure 5:**
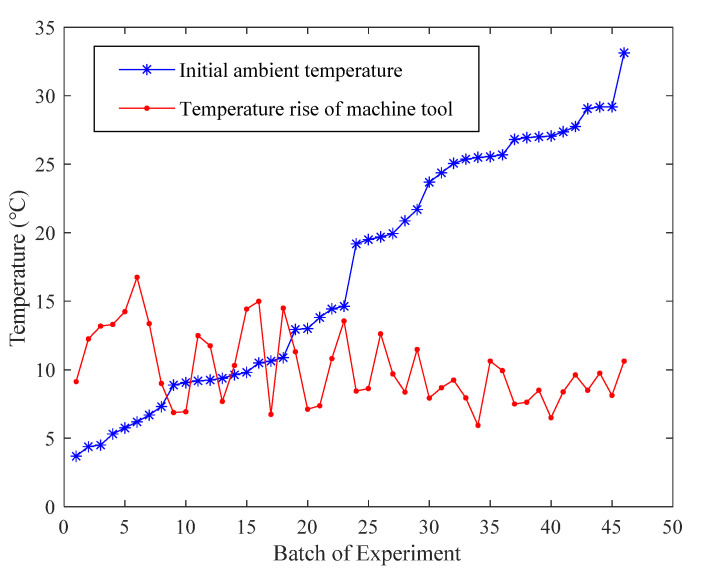
Statistical results of temperature changes of 46 batches of experiments.

According to the exploratory analysis of the experimental data, the initial ambient temperature range was 4.1–32.2 °C, and the range of temperature increase in the machine tool was 5.9–16.8 °C. Figure 5 illustrates the temperature results of each batch of experimental data. 

### 3.3. Significance Analysis of Thermal Error Data

ANOVA [30] is mainly used to test the significance of the difference between the mean values of two or more samples. According to the number of influencing factors, it includes one-way, two-way, and multi-factors ANOVA. We used one-way ANOVA to test whether the ambient temperature had a significant influence on the thermal error variation law of the machine tool.

If we assume that there are r batches of thermal error experimental data under different ambient temperatures and n thermal error measurement data in each batch of the experiment, then all the thermal error experimental data would be recorded as $y_{ij}\left(i=1,\;2,\ldots ,r;j=1,\;2,\ldots ,n\right)$. The thermal error average value of each batch was ${\bar{y}}_{i}=\sum_{j=1}^{j=n}y_{ij}$, and the thermal error average value of all batches was $\bar{y}=\sum_{i=1}^{i=r}{\bar{y}}_{i}$. The basic steps of one-way ANOVA are as follows.

1. Calculation of test statistics. The calculation formulas of the sum of squares of the total deviation $S_{T}$, the sum of squares of the intra-group deviation $S_{E}$, and the sum of squares of the inter-group deviations $S_{A}$, respectively, are shown below.

(11) $$\begin{array}{c} \left\{\begin{array}{c} S_{T}=\overset{i=r}{\underset{i=1}{\sum }}\overset{j=n}{\underset{j=1}{\sum }}{\left(y_{ij}-\bar{y}\right)}^{2}\\ S_{E}=\overset{i=r}{\underset{i=1}{\sum }}\overset{j=n}{\underset{j=1}{\sum }}{\left(y_{ij}-{\bar{y}}_{i}\right)}^{2}\\ S_{A}=n\overset{i=r}{\underset{i=1}{\sum }}{\left({\bar{y}}_{i}-\bar{y}\right)}^{2} \end{array}\right. \end{array}$$

The degrees of freedom (DoF) of the three statistical parameters are $df_{T}=n*r-1$, $df_{E}=n*r-r$, and $df_{A}=r-1$.

2. *F*-test statistics. The statistics in step (1) were divided by the corresponding DoF to calculate the mean square of the sum of squares to eliminate the interference caused by different DoF. This will be conducive to the comparison of the sum of squares of the deviations of each group of data. Then, the ratio F of the average of the squares of the inter-group deviations $S_{A}$ to the average of the squares of intra-group deviations $S_{E}$ was used to test the original hypothesis.

(12) $$\begin{array}{c} F=\frac{S_{A}/df_{A}}{S_{E}/df_{E}} \end{array}$$

3. Determine the significance. Test statistic F follows the F distribution with $df_{A}$ and $df_{E}$. The larger the value of F, the more inclined it is to reject the original hypothesis. Therefore, the rejection domain of the test is as follows:

(13) $$\begin{array}{c} W=\left\{F\geq F_{1-\alpha }\left(df_{A},df_{E}\right)\right\}\# \end{array}$$

where α is the significance level, and $F_{1-\alpha }\left(df_{A},df_{E}\right)$ is calculated through the F distribution table. If $F\geq F_{1-\alpha }\left(df_{A},df_{E}\right)$, the thermal error data of each batch have significant differences. Otherwise, the difference is not significant.

The p value of the test is obtained by the density function of the F distribution.

(14) $$\begin{array}{c} p=P\left(Y\geq F\right) \end{array}$$

In general, if p≤0.001, each batch of thermal error data is considered to be significantly different; if p≥0.05, the difference is not significant.

One-way ANOVA for the thermal error data in the Z direction of each batch was performed following the aforementioned steps, and the results are summarized in Table 2. In Table 2, $S_{s}$ is the sum of squares of deviations, $d_{f}$ is the DoF, $M_{s}$ is the mean square, *F* is the statistic to be tested, *p*-value is the density function p of the statistic F to be tested in the *F* distribution, and *F_crit_* is the critical value of the F statistic when the confidence level α = 0.001.

According to the analysis results in Table 2, the value of F is significantly larger than the critical value *F_crit_* of 1.79, with a DoF of (46, 3036), at the confidence level of p = 0.001. Meanwhile, the *p*-value corresponding to F is close to 0, which is markedly less than 0.001, indicating that the ambient temperature has a very significant impact on the thermal error data.

### 3.4. Analysis of Mutual Prediction Results with Annual Experimental Data

Each of the 46 batches of experiments was used to establish a TEPMs and predict the remaining batches of experiments. Thus, the mutual prediction results of 46 batches of experiments can be obtained. First, TSPs were selected for each batch of experiments with the use of the TSP selection algorithm presented in Section 2.2. Two TSPs are selected for each batch of experiments in this study with reference to [18]. The experimental data of K1 batch are taken as an example to illustrate TMP selection. The correlation coefficient between the thermal error and each temperature variable is calculated by Equation (8), and the results are shown in Table 3.

It can be observed from Table 3 that the correlation coefficients of $T_{1}$ and $T_{5}$ are the largest, both of which are 0.92. Thus, *T*_1_ and *T*_5_ are selected as the TSPs, which are used to measure the heating of the spindle motor. In the same manner, the TSPs of each batch of experiments are selected, as listed in Table 4.

Based on the TSPs selection results, the TEPM of each batch of experiments was established using the PCR algorithm in Section 2.1. For example, the TEPMs of K1 and K46 are as follows.

(15) $$\begin{array}{c} Z_{1}=1.8767+1.8147T_{5}+4.9211T_{1} \end{array}$$

(16) $$\begin{array}{c} Z_{46}=2.1280+2.3108T_{1}+9.6814T_{5} \end{array}$$

Furthermore, the prediction results of each TEPM were calculated. The predicted residual standard deviation (Rsd) was used to represent prediction accuracy. The formula of the predicted Rsd is as follows:

(17) $$\begin{array}{c} Rsd=\sqrt{\frac{\sum_{j=1}^{n}{\left(\hat{y_{j}}-y_{j}\right)}^{2}}{n-1}}, \end{array}$$

where $y_{j}$ is the jth measured thermal error value, and $\hat{y_{j}}$ is the jth predicted thermal error value.

**Figure 6:**
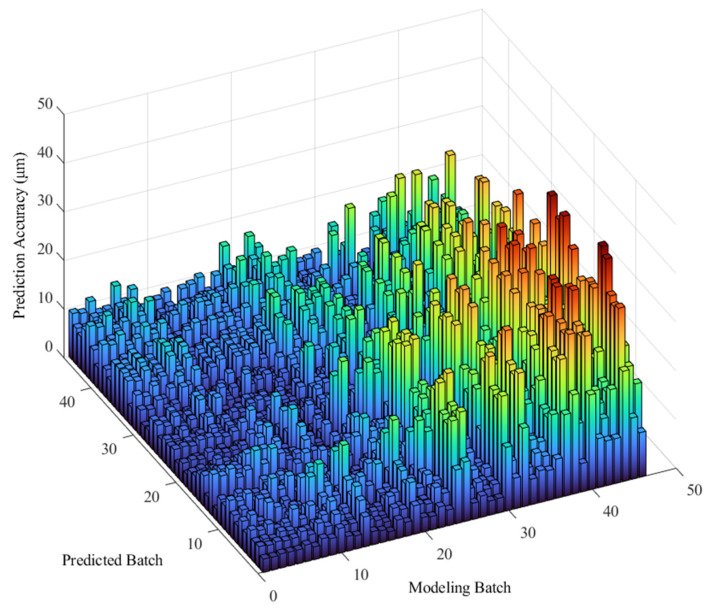
Mutual prediction results of 46 batches of experiments.

Finally, the mutual prediction results of the 46 batches of experiments were calculated and are presented in Figure 6, depicting the differences.

The mutual prediction accuracy is high when the ambient temperature is within a certain range, as shown in Figure 6. However, when the ambient temperature exceeded a certain range, prediction accuracy decreased; i.e., the robustness decreased. In addition, the model based on experimental data under low ambient temperature has a better prediction effect than the one based on the high-temperature data. 

**Figure 7:**
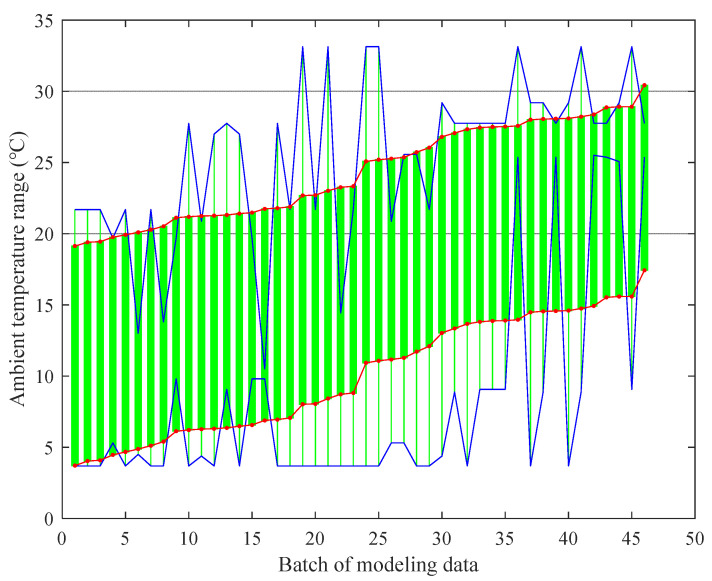
Applicable ATIs of TEPMs of 46 batches of experiments.

When using a TEPM to predict the thermal error data under different ambient temperatures, the method can only maintain high prediction accuracy for data within a certain ambient temperature range. We call this temperature range the “applicable ATI of TEPM”. For each TEPM, experiment batches with an *Rsd* of less than 5 μm were grouped according to the mutual prediction results presented in Figure 6. Then, the ambient temperature range of these experiment batches was obtained as the applicable ATI of the prediction model. Thus, the applicable ATI of the TEPM of experimental data from each batch was obtained (Figure 7). The horizontal axis in the figure is the batch of the modeling data. The vertical axis is the ambient temperature range. The blue dotted lines are the upper and lower boundaries of all applicable ATIs. The upper and lower boundaries of the ATIs were fitted as the red lines.

The applicable ATIs fluctuated by a margin due to some random factors, such as measurement error. It is obvious that the established TEPMs have different applicable ATIs. This proves the necessity of the proposed modeling method based on ATIs for compensation through an entire year.

## 4. Thermal Error Modeling and Compensation Method Based on ATIs

To solve the problem of decreased thermal error prediction effects over the annual ambient temperature range, we proposed a year-round thermal error modeling and compensation method based on ATIs. In this section, an overview of the proposed modeling and compensation method is first introduced in Section 4.1. In Section 4.2, the ATIs are determined using the C-Means clustering algorithm and the optimal number of ATIs is obtained based on the prediction results of TEPMs corresponding to the number of ATIs. Finally, the prediction effects of the established models are analyzed based on the experimental data collected over one year in Section 4.3.

### 4.1. Overview of the Proposed Method Based on ATIs

As the TEPM established by the experimental data under a fixed ambient temperature can only maintain high prediction accuracy in its applicable ATI, it is necessary to establish the corresponding TEPMs for different ambient temperature ranges, namely the modeling and compensation method based on ATIs.

**Figure 8:**
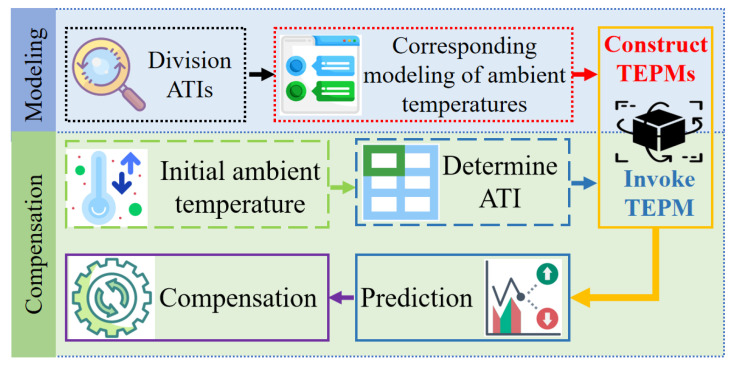
Flowchart of the modeling and compensation method based on ATIs.

Figure 8 presents the flowchart of the proposed method that contains two processes. First, in the modeling process, ATIs are divided and the ambient temperature corresponding to each ATI is determined based on the C-Means clustering algorithm. Subsequently, TEPMs are constructed for each determined ATI. In the compensation process, ATI would be determined according to the initial ambient temperature value. Then, the corresponding TEPM is invoked to predict the thermal errors. Finally, thermal error compensation would be performed according to the predicted value. Thus, the high prediction accuracy and robustness can be realized for the thermal error within the annual ambient temperature range.

### 4.2. Determination of the ATIs

The key to the success of the proposed modeling and compensation method is determining ATIs reasonably. To achieve that, the C-Means clustering algorithm is utilized to determine the ATIs. As the number of ATIs is not unclear, the prediction effects are analyzed to obtain the optimal number of ATIs. In this subsection, the C-Means clustering algorithm is first introduced and then the optimal number of ATIs is analyzed and obtained.

#### 4.2.1. C-Means Clustering Algorithm

C-Means clustering is a typical algorithm for dynamic clustering. The ambient temperature variables of 46 batches of experiments are clustered using the C-Means clustering algorithm. According to the clustering results, the initial ambient temperature variation range of the ambient temperature variables of the same class is considered as one ATI. The basic steps of dividing the ATIs based on the C-means clustering algorithm are as follows.

1. Given the number of clusters M and randomly selecting M initial cluster centers $C_{1},\;C_{2},\ldots ,C_{M}$

2. Calculate the distance from each ambient temperature variable to each cluster center, and the calculation formula is as shown:

(18) $$\begin{array}{c} d\left(x,C\right)=\overset{n}{\underset{k=1}{\sum }}\left|x_{k}-C_{k}\right|, \end{array}$$

where x  represents the ambient temperature variable, and n represents the number of sample data in temperature variable.

3. Divide the ambient temperature variables closest to the cluster center into this cluster;
4. Recalculate cluster center according to the clustering results, that is, take the average value of the ambient temperature variables belonging to the same cluster as the new cluster center;
5. Repeat steps 2 to 4 until the cluster results remains unchanged or the maximum number of iterations is reached;
6. Consider the initial ambient temperature range contained in the same class as one ATI.

For example, suppose the number of clusters is 4; then, the 46 ambient temperature variables of the 46 batches of experiments are grouped into four classes, as shown in Table 5. The demarcation of the two adjacent ATIs is calculated by averaging the boundaries of the two adjacent initial ambient temperature ranges. For example, the demarcation of the first and second ATI is (7.0 + 9.1)/2 ≈ 8.1.

The TEPM established by the experimental data at the ambient temperature at the left end of the ATI is used as the TEPM of the ATI. In this way, the TEPMs corresponding to the four ATIs in Table 5 can be obtained, as shown below.

(19) $$\begin{array}{c} \left\{\begin{array}{c} Y_{1}=1.8767+1.8147T_{5}+4.9211T_{1}\;4.1\leq T_{0}\leq 8.1\\ Y_{9}=2.2417+2.2222T_{1}+3.4832T_{5}\;8.1<T_{0}\leq 17.2\\ Y_{24}=1.8492+1.8419T_{1}+7.2712T_{5}\;17.2<T_{0}\leq 24.0\\ Y_{31}=2.6280+2.7416T_{1}+0.2192T_{5}\;24.0<T_{0}\leq 32.2 \end{array}\right. \end{array}$$

The coefficients in the regression models, as shown in Equation (19), are all positive, which show that when the temperature rises, the thermal error increases as well. This is consistent with the physical law in general. It is also noteworthy that all 46 batches of data (only temperature information) are used as initial batches for determining the ATIs. In practice, a general rule to reduce the number of initial batches is that it is suitable to reduce the batches when the batches of experiments are conducted within a similar ambient temperature interval, but reducing them is not suitable when they are conducted with significant different ambient temperatures. As a result, the number of initial batches for determining the ATIs can be reduced while the information contained in the data is not lost. In addition, the number of initial batches (both temperature and thermal error information) for modeling is the same as the number of ATIs, and the effects of different numbers of ATIs on the performance will be investigated in the next subsection.

#### 4.2.2. Modeling Effect Analysis of Different Numbers of ATIs

The number of ATIs needs to be given before dividing the ATIs using the C-Means clustering algorithm. Thus, the number of ATIs has an important influence on the prediction effects. In order to obtain the optimal number of ATIs, the TEPMs of different ATIs are established. Then, the established TEPMs are used to predict the experimental data of an entire year. According to the prediction effects, the optimal number of ATIs can be obtained using the elbow method. 

As for the established TEPMs of ATIs, the mean and standard deviations [31] of the Rsd of the 46 batches of experiments can be calculated as follows:

(20) $$\begin{array}{c} S_{M}=\frac{1}{K}\overset{K}{\underset{k=1}{\sum }}Rsd_{k}, \end{array}$$

(21) $$\begin{array}{c} S_{D}=\sqrt{\frac{\sum_{k=1}^{K}{\left(Rsd_{k}-S_{M}\right)}^{2}}{K-1}}, \end{array}$$

where $Rsd_{k}$ is the Rsd value of the kth batch of experiments, and they can be calculated by Equation (17). K=46 is the total number of batches of the predicted experiments. $S_{M}$ and $S_{D}$ are used to characterize the prediction accuracy and robustness of the model.

**Figure 9:**
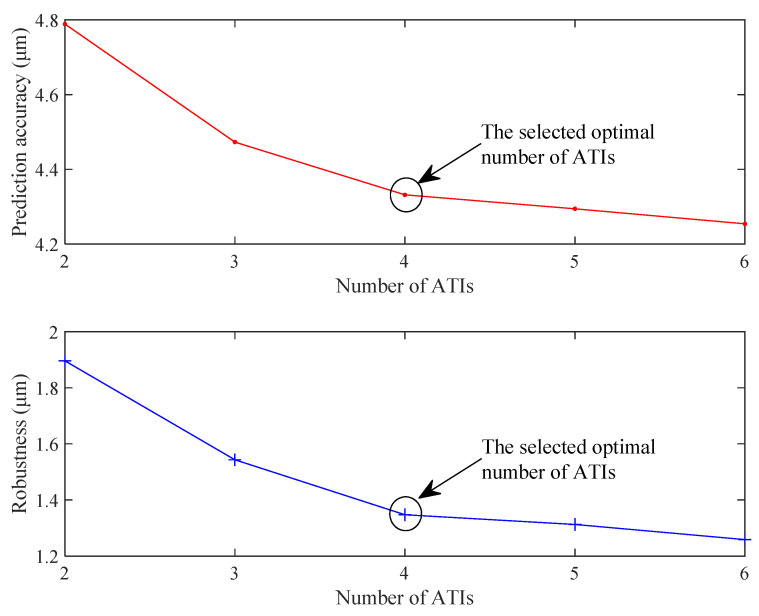
Prediction effects with different numbers of ATIs.

In this study, the number of ATIs ranges from 2 to 6 with a step size of 1. Then, the prediction accuracy and robustness with different number of ATIs are calculated using Equations (20) and (21). The calculation results are plotted in Figure 9 to visually show the influence of the number of ATIs on the prediction effects. It can be observed from Figure 9 that the prediction effects gradually improved as the number of ATIs increases. Marked by the black circle in Figure 9, when the ATIs reaches four, the prediction effects gradually stabilize. Therefore, the number of ATIs is determined as four according to the elbow method. As a side note, the method in [25] with one segmentation point is only one special case of our generic method when the number of ATIs is two.

### 4.3. Prediction Performance Analysis with Annual Experimental Data

**Figure 10:**
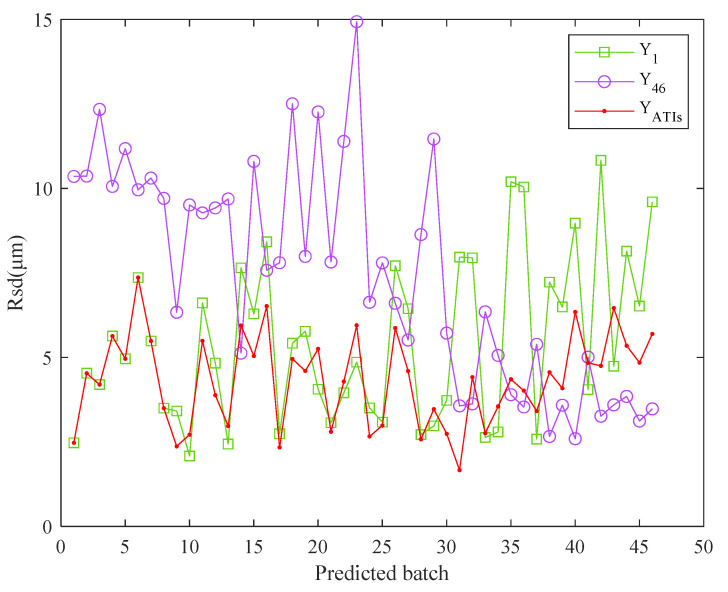
Prediction results of the three models for the annual experiments.

The TEPMs corresponding to 4 ATIs are shown in Equation (19), which are used to predict the annual thermal error data. The low and high ambient temperature models $Y_{1}$ and $Y_{46}$ were also used to predict the annual thermal error data. The prediction results Rsd of these three models were calculated and plotted in Figure 10. Note that Figure 10 is plotted based on 46 batches of data, which were collected over an entire year, as mentioned before.

As shown in Figure 10, the ATI model has higher prediction accuracy than the low and high ambient temperature models. This is because the proposed year-round thermal error modeling method not only takes the ATIs into consideration but also determines the optimal number of ATIs to achieve better performance, which is a key difference from the existing literature. Furthermore, the prediction results of three models are summarized in Table 6. Table 6 reveals that the TEPMs established based on the ATIs exhibit high prediction accuracy and robustness throughout the year. Further calculation showed that the prediction accuracy of the proposed ATI model was 20.6% and 41.7% higher than those of the $Y_{1}$ and $Y_{46}$, respectively, and robustness was improved by 48.8% and 62.0%, respectively. In the thermal error compensation process, the TEPM corresponding to the ATI could be invoked for prediction and compensation, according to the actual initial ambient temperature.

Mean of Rsd Standard deviation of Rsd

## 5. Conclusions

In this study, the one-way ANOVA was first conducted to show that the ambient temperature has a significant impact on the spindle thermal error data at a significant level of 0.001. Therefore, a thermal error modeling and compensation method for spindle of machine tool based on ATIs was proposed in this paper. Based on the annual experimental data, the ATIs applicable to the TEPMs were investigated, and the number of ATIs based on the C-Means clustering algorithm was determined as 4. The prediction accuracy and robustness of the proposed method were 4.33 μm and 1.24 μm, respectively, demonstrating the high prediction accuracy and robustness of the method throughout the year. In addition, the prediction accuracy of the proposed ATI model was 20.6% and 41.7% higher than those of the $Y_{1}$ and $Y_{46}$, respectively, and robustness improved by 48.8% and 62.0%, respectively. This study can serve as an important reference for machine tools of thermal error modeling and compensation.

Obtaining the applicable ATIs is the basis of the proposed method. In fact, the number of ATIs is related to the range of the applicable ATI of the TEPM, which must be determined according to the actual scenario. Factors affecting the range of the applicable ATI, such as the modeling algorithm and the machine tool itself, should be the focus of future research.

## Figures and Tables

**Table 1:** Installation sites and measurement functions of temperature sensors.

Sensors	Installation Site	Function
T1~T5	Front bearing of Spindle	Bearing temperature measurement
T7,T8	Spindle motor	Spindle motor temperature measurement
T6,T9	Spindle box	Spindle box temperature measurement
T10	Machine frame	Ambient temperature measurement

**Table 2:** ANOVA and significance test results.

Source of Difference	$S_{s}$	$d_{f}$	$M_{s}$	F	*p*-Value	*F_crit_*
Type	388,844	46	8640.978	113.5396	Close to 0	1.790356
Error	231,055.9	3036	76.10538			
Total	619,900	3081				

**Table 3:** Correlation coefficient between temperature variable and thermal error of K1.

Sensors	$T_{1}$	$T_{2}$	$T_{3}$	$T_{4}$	$T_{5}$	$T_{6}$	$T_{7}$	$T_{8}$	$T_{9}$	$T_{10}$
Correlation coefficient	0.92	0.92	0.91	0.92	0.92	0.85	0.77	0.86	0.86	0.70

**Table 4:** Selection results of TSPs for each batch.

Batch	K1	K2	K3	K4	K5	K6	K7	K8	K9	K10
Results	T1, T5	T1, T5	T1, T3	T1, T5	T1, T5	T1, T4	T1, T2	T1, T4	T1, T5	T1, T5
Batch	…	K38	K39	K40	K41	K42	K43	K44	K45	K46
Results	…	T1, T8	T1, T5	T1, T8	T1, T5	T1, T5	T1, T5	T8, T9	T1, T5	T1, T5

**Table 5:** Clustering results with four clusters.

Class	Results	Initial Ambient Temperature Range	ATI
Class 1	1–8	4.1–7.0	[4.1–8.1]
Class 2	9–23	9.1–14.5	(8.1–17.2]
Class 3	24–30	19.8–23.4	(17.2–24.0]
Class 4	31–46	24.6–32.2	(24.0–32.2]

**Table 6:** Prediction results of the three models (Unit: μm).

Model	ATI Model	$Y_{1}$	$Y_{46}$
Mean of Rsd	4.33	5.45	7.43
Standard deviation of Rsd	1.24	2.42	3.26

## Data Availability

Not applicable.
